# Pathogens transmitted in animal feces in low- and middle-income countries

**DOI:** 10.1016/j.ijheh.2018.03.005

**Published:** 2018-05

**Authors:** Miranda J. Delahoy, Breanna Wodnik, Lydia McAliley, Gauthami Penakalapati, Jenna Swarthout, Matthew C. Freeman, Karen Levy

**Affiliations:** Department of Environmental Health, Emory University Rollins School of Public Health, 1518 Clifton Road NE, Atlanta, GA 30322, USA

**Keywords:** Zoonotic pathogens, Animal feces, Diarrhea, Enteropathogens, Water sanitation & hygiene

## Abstract

Animals found in close proximity to humans in low-and middle-income countries (LMICs) harbor many pathogens capable of infecting humans, transmissible via their feces. Contact with animal feces poses a currently unquantified—though likely substantial—risk to human health. In LMIC settings, human exposure to animal feces may explain some of the limited success of recent water, sanitation, and hygiene interventions that have focused on limiting exposure to human excreta, with less attention to containing animal feces.

We conducted a review to identify pathogens that may substantially contribute to the global burden of disease in humans through their spread in animal feces in the domestic environment in LMICs. Of the 65 potentially pathogenic organisms considered, 15 were deemed relevant, based on burden of disease and potential for zoonotic transmission. Of these, five were considered of highest concern based on a substantial burden of disease for which transmission in animal feces is potentially important: *Campylobacter,* non-typhoidal *Salmonella* (NTS), Lassa virus, *Cryptosporidium*, and *Toxoplasma gondii*. Most of these have a wide range of animal hosts, except Lassa virus, which is spread through the feces of rats indigenous to sub-Saharan Africa. Combined, these five pathogens cause close to one million deaths annually. More than half of these deaths are attributed to invasive NTS. We do not estimate an overall burden of disease from improperly managed animal feces in LMICs, because it is unknown what proportion of illnesses caused by these pathogens can be attributed to contact with animal feces.

Typical water quantity, water quality, and handwashing interventions promoted in public health and development address transmission routes for both human and animal feces; however, sanitation interventions typically focus on containing human waste, often neglecting the residual burden of disease from pathogens transmitted via animal feces. This review compiles evidence on which pathogens may contribute to the burden of disease through transmission in animal feces; these data will help prioritize intervention types and regions that could most benefit from interventions aimed at reducing human contact with animal feces.

## Highlights

• We reviewed pathogens transmitted in animal feces in low-/middle-income countries.
• Five pathogens of highest concern cause approximately one million annual deaths.
• The proportion of deaths attributable to contact with animal feces remains unknown.
• This review can help prioritize interventions and regions for animal feces control.

## 1 Introduction

Many pathogens capable of infecting humans can be found in animal feces, yet the feces from these animals pose a currently unquantified—though likely substantial—risk to human health (Dufour et al., 2012). Insufficient separation of animal feces from human domestic environments can lead to fecal-oral transmission of zoonotic pathogens through direct contact with animal feces or soil, or fecal contamination of fomites, food, or water sources (Penakalapati et al., 2017). In addition to the acute gastrointestinal symptoms that can arise from contact with animal feces, people—particularly children, pregnant women, and the immunocompromised—may experience severe sequelae after a zoonotic infection (Checkley et al., 1998; Dufour et al., 2012). There is currently no estimate of the burden of poorly managed animal feces on human health.

Systematic reviews have demonstrated a 30–40% decrease in childhood diarrhea after the introduction of improved sanitation in low- and middle-income countries (LMICs) (Freeman et al., 2017; Wolf et al., 2014), but several recent water, sanitation, and/or hygiene interventions have failed to find consistent evidence of decreasing rates of childhood diarrhea, decreasing fecal contamination of household stored water, decreasing soil-transmitted helminth (STH) infections, and/or improving anthropometric indicators of malnutrition (Clasen et al., 2014; Null et al., 2018; Patil et al., 2014). One possible conclusion is that these interventions failed to interrupt a critical pathway in pathogen transmission: exposure to animal feces.

Improvements to water quality, water quantity, and handwashing could reduce exposure to both human and animal pathogens. However, providing access to improved sanitation by definition involves separating humans from contact with their own excreta, but not from animal waste (WHO/UNICEF, 2015). Less attention has been given to the role of contact with animal feces in causing human illness (Dufour et al., 2012). Animals are often present in the domestic environment in LMICs, and people in these countries may have frequent contact with them (Dione et al., 2011; Harvey et al., 2003; Zambrano et al., 2014). Thus, even as sanitation efforts may reduce the quantity of human excreta in the environment, contamination from animal feces may still contribute to substantial burden of disease in humans.

Understanding the burden of disease from animal feces would provide critical information to direct public health investments, yet consolidated information on which pathogens may be transmitted in animal feces is lacking, making it difficult to assess the importance of animal feces exposure to human health. In this review, we identify pathogens that may substantially contribute to the global burden of disease in humans through their spread in animal feces in the domestic (household) environment in LMICs, and identify the animals that transmit these pathogens. Understanding the contribution of animal feces to the burden of disease involves identifying pathogens that can be transmitted in animal feces, understanding the extent of zoonotic transmission, and establishing that such zoonotic transmission can give rise to illness in humans. This review accumulates evidence on these topics from recent research in LMICs.

## 2 Methods

### 2.1 Pathogen inclusion criteria

We evaluated bacteria, protozoa, viruses, and helminths for inclusion in this review using a multistep process. As the majority of reviewed literature on human health impacts from exposure to animal feces has focused on health outcomes related to exposure to enteric pathogens or helminths (including both short- and long-term sequelae such as diarrhea and malnutrition) (Penakalapati et al., 2017), we first considered all enteric pathogens and STHs that were included in two recent large studies describing an array of enteric pathogens/STHs: the Global Enteric Multicenter Study (GEMS) and MAL-ED (The Etiology, Risk Factors, and Interactions of Enteric Infections and Malnutrition and the Consequences for Child Health Study) (Houpt et al., 2014; Panchalingam et al., 2012). Animal feces also pose a risk to human health by contaminating water sources (Dufour et al., 2012). Thus, we next considered waterborne excreted pathogens listed in the Global Water Pathogen Project as of August 29, 2017 (Rose and Jiménez-Cisneros, 2017), which provides an extensive compilation of waterborne pathogens. Third, we identified potential pathogens through our systematic review of the health impacts of exposure to poorly managed animal feces (see Penakalapati et al., 2017 for detailed methods), as this review was specifically focused on health outcomes (including pathogen infection) resulting from exposure to animal feces, and was not restricted to literature on waterborne or enteric pathogens. We considered pathogens detected in studies that met the inclusion criteria of that review, as well as pathogens from additional papers marked as relevant during the title and abstract assessment, specifically if the abstract mentioned pathogens not previously considered or new pathogen-animal host pairs from research in an LMIC.

Pathogens were categorized based on burden of disease and potential for transmission in animal feces. The transmission of pathogens via animal feces in LMICs was considered “potentially important” if (1) most transmission to humans resulted from exposure to the feces of specific animals, rather than human feces (e.g., rats for Lassa virus) or (2) the feces of a broad range of animal hosts pose a risk of transmitting the pathogen and causing illness. Transmission via animal feces in LMICs was considered to be “of limited importance, or insufficient evidence of importance” for pathogens that (1) were predominantly transmitted from human-to-human and, while transmitted in animal feces, did not have a wide range of animal hosts or (2) had insufficient epidemiologic and/or molecular evidence of contributing to the human disease burden through zoonotic transmission. If zoonotic species of an organism were less pathogenic to humans than anthropogenic strains (and thus zoonotic strains were not thought to account for a large proportion of the symptomatic illness caused by that pathogen), the role of animal fecal transmission in the burden of disease was also considered “of limited importance, or insufficient evidence of importance”.

Pathogens had to meet the following criteria to be included. Exclusion criteria are noted where applicable:

1. **Found in animal feces in domestic settings.** Species of the pathogen capable of infecting humans can be found in the feces of animals that are common to domestic settings. Pathogens that are only transmitted by a small number of wild animal species (e.g., pathogens transmitted only by primates) or aquatic species were excluded.
2. **Cause illness in humans.** The pathogen is linked to illness in humans. Pathogens that primarily cause illness in animals with humans serving only as incidental dead-end hosts were excluded. Organisms generally considered commensals that occasionally give rise to sporadic opportunistic infections were excluded.
3. **Substantial contribution to disease burden.** The pathogen contributes substantially to the global burden of disease in humans. We considered pathogens responsible for either a minimum of one million disability-adjusted life years (DALYs), or at least 5,000 deaths annually to have a “substantial” contribution to the burden of disease in humans. Pathogens with an unquantified burden of disease were included, whereas pathogens with a quantified burden of disease that was not “substantial” were excluded.

Once identified for inclusion, pathogens were classified into the following categories. Category I pathogens contribute substantially to the burden of disease and the role of animal feces in transmission is potentially important. Category II pathogens have an unquantified burden of disease, and the role of animal feces is also potentially important. Category III and Category IV pathogens have limited or insufficient evidence of transmission via animal feces. Category III pathogens have a substantial burden of disease, whereas the burden for Category IV pathogens is unquantified.

### 2.2 Literature review extraction

Information for this review was gathered from our team's literature review of the impact of animal feces on human health (Penakalapati et al., 2017), reference texts (Heymann and American Public Health Association, 2015; Rose and Jiménez-Cisneros, 2017), and from targeted journal searches. For the included pathogens, all literature uncovered in our review of the impact of animal feces on human health was included if it reported an association between exposure to animals or animal feces and the detection of a human pathogen infection. Information on burden of disease was primarily collected from the mortality analysis of the 2015 Global Burden of Disease Study (GBD) (Wang et al., 2016); however, targeted searches were used to assess burden for pathogens not included in the GBD.

For Category I pathogens, which were considered the pathogens of highest concern, we extracted information on the burden of disease, clinical manifestation, known animal hosts, transmission routes/burden associated with zoonotic transmission, relevant species, control options proposed in existing literature, and other information relevant to host susceptibility and transmission. Shorter descriptions of Category II–IV pathogens covering similar topics are also included. For excluded pathogens, we documented references to support exclusion. Proposed control options and other information on host susceptibility and transmission were included when identified in the literature we reviewed, but are not ordered based on any specific criteria and do not constitute a comprehensive list. While the transmission of antimicrobial resistant bacteria may be of concern when considering exposure to animal feces, it was not specifically a focus of this review.

## 3 Results

**Fig. 1:**
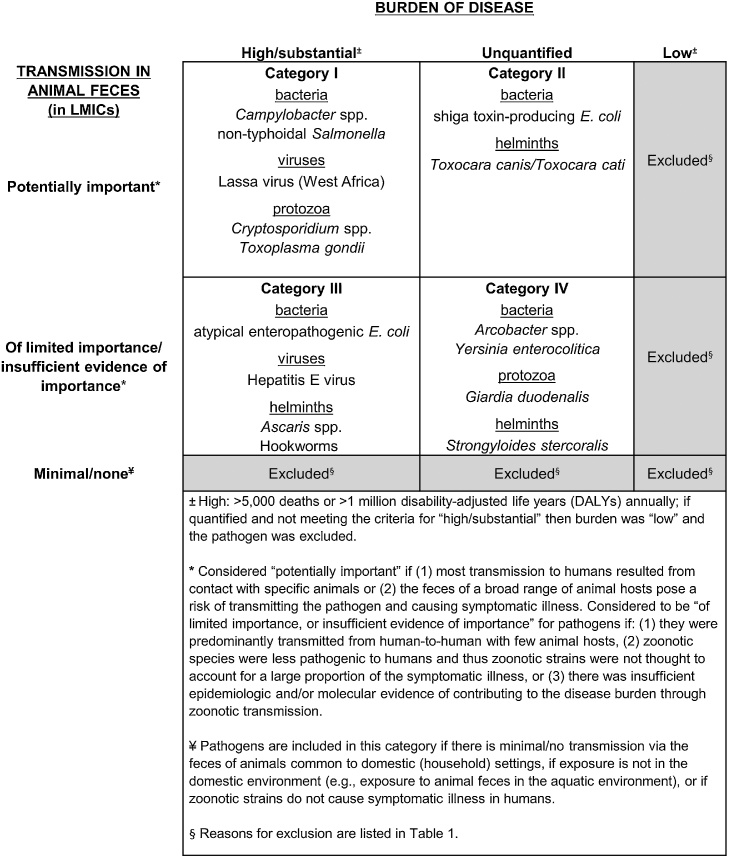
Classification of pathogens by burden of disease and potential for transmission in animal feces in domestic/household settings in low- and middle-income countries (LMICs).

Approximately 65 potentially pathogenic species or groups (“potential pathogens”) were considered for inclusion (Fig. 1 and Table 1). The exact number of potential pathogens depends on how they are grouped; for example, several different *Escherichia coli* pathotypes were counted as distinct pathogens. We excluded 50 potential pathogens, and outline the reasons for these exclusions in Table 1. Classification grouping of the remaining 15 pathogens is shown in Fig. 1 and discussed below for each category, ordered by bacteria, viruses, protozoa, then helminths. The five pathogens of highest concern (Category I) are *Campylobacter,* non-typhoidal *Salmonella*, Lassa virus, *Cryptosporidium*, and *Toxoplasma gondii*.

**Table 1:** Reasons for excluding potential pathogens from the list of pathogens that potentially substantially contribute to the burden of disease via transmission in animal feces in the household setting in low-and middle-income countries.

Potential pathogen	Exclusion reason^a^
**Bacteria**	
*Aeromonas* spp.	Reservoirs are primarily humans and aquatic environments/organisms (Janda et al., 1983); pathogen has been isolated from other animals but not linked to human health outcomes (Parker and Shaw, 2011)
*Brucella* spp.	Most zoonotic transmission occurs when humans assist in birthing animals, or from eating unpasteurized milk products; these bacteria do not survive well in the environment; while animal contact may be an important risk factor, exposure to animal feces in the domestic setting does not seem to be an established exposure route to humans (Atluri et al., 2011)
*Chlamydia trachomatis*	Transmitted human-to-human via *Musca sorbens*; while animal feces may provide a breeding area for this vector, the preferred breeding area is human feces (Emerson et al., 2001); exposure to animal feces in the household not considered the transmission route
Diffusely adherent *E. coli* (DAEC)	Hosts unknown and may not include animals (Croxen et al., 2013)
Enteroaggregative *E. coli* (EAEC)	Isolated in animals but this is not thought to be a source of human infections (Okhuysen and DuPont, 2010)
Enteroinvasive *E. coli* (EIEC)	Limited animal hosts (primates) (Croxen et al., 2013)
Enterotoxogenic *E. coli* (ETEC)	Zoonotic strains not pathogenic to humans: adhesion factors are species-specific (Torres, 2010; Wasteson, 2002)
*Helicobacter pylori*	Generally human-to-human transmission; the role of animals and food is controversial (Safaei et al., 2011; Vale and Vítor, 2010)
*Klebsiella* spp.	Generally considered commensals, but may cause opportunistic infections in the immunocompromised; however, given low pathogenicity unlikely to contribute substantially to burden of disease (Ristuccia and Cunha, 1984)
*Leptospira* spp.	Transmitted in urine (Bharti et al., 2003)
*Mycobacterium avium* subspecies *paratuberculosis*	May contribute to Crohn’s disease (the burden of which is largely unknown in developing countries), but link to human illness is not well-defined (Economou and Pappas, 2008)
*Plesiomonas shigelloides*	Animal feces not implicated as transmission route; non-human hosts are mostly aquatic animals (Janda et al., 2016)
*Salmonella paratyphi*	Not transmitted in animal feces (Crump et al., 2015; Gal-Mor et al., 2014)
*Salmonella typhi*	Not transmitted in animal feces (Crump et al., 2015; Gal-Mor et al., 2014)
*Shigella* spp.	Limited animal hosts (primates) (Heymann and American Public Health Association, 2015)
*Vibrio cholerae*	Limited/no transmission by animal feces (Heymann and American Public Health Association, 2015; Nelson et al., 2009)
*Yersinia pestis*	Transmitted to humans through flea bites (Achtman et al., 1999)
**Viruses**	
Astrovirus	No documented cases of zoonotic transmission (De Benedictis et al., 2011)
Enteric adenovirus 40/41	Enteric adenovirus strains 40/41 are human-specific (Ghebremedhin, 2014)
Hepatitis A virus	Limited animal hosts (primates) (Heymann and American Public Health Association, 2015)
Human enteroviruses (A–D)	Not considered zoonotic (Taylor et al., 2001); includes polioviruses, Coxsackievirus, and echoviruses
Human papillomaviruses	Human viruses that are predominately transmitted sexually or from mother to child (Syrjanen and Puranen, 2000)
Norovirus (GI/GII)	Humans are known hosts of norovirus GI/GII (Glass et al., 2009)
Picobirnavirus	Human pathogenicity is debated; little evidence of cross-species transmission (Ganesh et al., 2012)
Polyomavirus	Human polyomaviruses are host-specific to humans (Bofill-Mas et al., 2013)
Rotavirus	Strains found in animals unlikely to infect humans (Cook, 2004)
Sapovirus	Has been isolated in swine feces but not a major route of transmission to humans, if a route at all (Bank-Wolf et al., 2010)
**Protozoa**	
*Balantidium coli*	Low prevalence in humans; low virulence; public health significance thought to be low as most cases are asymptomatic (Ponce-Gordo and Jirků-Pomajbíková, 2017; Schuster and Ramirez-Avila, 2008)
*Cyclospora cayetanensis*	Not transmitted in animal feces (Mansfield and Gajadhar, 2004)
*Endolimax nana*	Pathogenicity and host specificity remain debated; likely a commensal in humans (Poulsen and Stensvold, 2016)
*Entamoeba* spp.	Zoonotic transmission not thought to contribute significantly to burden of disease (Thompson and Smith, 2011)
*Trichomonas hominis*	Low pathogenicity; unlikely to contribute significantly to the burden of disease (Li et al., 2015; Rüttgers, 1983)
**Helminths**	
*Ascaridia galli*	Pathogenic to poultry, not humans (Höglund and Jansson, 2011)
*Clonorchis sinensis, Metorchis* spp.*, Opisthorchis* spp. (human liver flukes)	Transmitted to humans by ingestion of fish (Murell and Pozio, 2016)
Diphyllobothriidea	Though some may be transmitted in animal feces, transmission to humans is associated with eating contaminated fish (Waeschenbach et al., 2017)
*Echinococcus granulosus/Echinococcus multilocularis*	Excluded based on burden of disease (<5000 deaths and <1 million disability-adjusted life years (DALYs) annually) (Kassebaum et al., 2016; Wang et al., 2016)
*Enterobius* spp.	*Enterobius vermicularis*, responsible for infections in humans, are human pinworms without non-primate animal hosts (Knopp et al., 2012)
*Fasciola* spp.	Exposure to aquatic environment and plants is a main source of transmission to humans, though there are mammals that can also harbor these pathogens; burden estimated as <1 million DALYs (Fürst et al., 2012)
Heterophyidae and Echinostomatidae	These intestinal flukes are mostly transmitted to humans through ingestion of fish (Traub and Dalsgaard, 2016)
*Oesophagostomum bifurcum*	Limited to primates; limited evidence of cross-species transmission (Gasser et al., 2005)
*Paragonimus* spp.	Humans become infected by ingesting crustaceans (Vélez et al., 2003)
*Schistosoma* spp.	There are many mammalian species that shed eggs in their feces that are important to the transmission cycle; however, humans become infected by coming into contact with cercariae in water, not by coming into contact with mammalian feces (Colley et al., 2014)
*Spirometra* spp.	There are animal hosts that can shed *Spirometra* in their feces but animal feces in the domestic environment is not the major source of transmission to humans (Liu et al., 2015)
*Taenia* spp.	The burden of disease from cysticercosis was not considered substantial (<5000 deaths and <1 million DALYs annually) (Kassebaum et al., 2016; Wang et al., 2016)
*Trichuris* spp.	*Trichuris* spp. tend to be host-specific with human infections acquired from other primates (Ghai et al., 2014); responsible for <1 million annual DALYs (Hotez et al., 2014)
**Other**	
*Blastocystis* spp.	Pathogenicity for humans is still debated (Tan, 2008)
*Colonic spirochetosis*	Pathogenicity for humans is still debated (Calderaro et al., 2007)
*Enterocytozoon bieneusi*/microsporidia	*E. bieneusi* and other microsporidia mostly infect immunocompromised hosts; pathogenicity for immunocompetent hosts is not well defined and scope may be limited; of note, most infections appear to be zoonotic in origin (Didier, 2005; Matos et al., 2012)
Isospora	Animals do not serve as hosts for *Isospora belli*, which is responsible for most human cases; opportunistic infection in immunocompromised (Lindsay et al., 1997; Pierce and Kirkpatrick, 2009)
Mycetoma	Group of fungal infections not transmitted in animal feces (though not all transmission routes are known) (Zijlstra et al., 2016)

a Details on inclusion/exclusion criteria are outlined in manuscript methods (Section 2.1).

Of the 62 papers included in our team’s literature review of the impact of animal feces on human health (Penakalapati et al., 2017), 15 reported an association between exposure to animals or animal feces and human infection with one of the pathogens included in this review. The findings from each paper are integrated into the following pathogen descriptions.

### 3.1 Category I: pathogens with a substantial burden of disease with potentially important transmission in animal feces

#### 3.1.1 *Campylobacter* spp.

In 2015, *Campylobacter* caused an estimated 37,500 deaths from acute diarrhea (“diarrhea deaths”) globally, most of these (30,900) in children under five years old (Wang et al., 2016). *Campylobacter* is endemic in Africa, Asia, and the Middle East, and has been found in approximately 5–25% of gastrointestinal cases across a number of studies in LMICs (Kaakoush et al., 2015). The incidence and prevalence of *Campylobacter* have increased globally in the last decade, though deaths have decreased (Kaakoush et al., 2015; Wang et al., 2016). MAL-ED identified *Campylobacter* as a significant contributor to community diarrhea in children under two years old (Platts-Mills et al., 2015).

The main symptoms of *Campylobacter* infection are fever, diarrhea, and abdominal pain (Butzler, 2004). There are several potential long-term sequelae of *Campylobacter* infections, including reactive arthritis and Guillain-Barré syndrome (Butzler, 2004; Wilson et al., 2008).

*Campylobacter* is most often found in poultry and cattle; other animals—including young dogs and cats, other pets, pigs, rodents, and birds—may also be reservoirs of *Campylobacter* capable of infecting humans (Heymann and American Public Health Association, 2015). *Campylobacter coli* and *C. jejuni* are the primary species of importance to human health; *Campylobacter concisus* and *Campylobacter ureolyticus* are emerging species that may also be important to human health (Kaakoush et al., 2015).

*Campylobacter* transmission occurs through foodborne and waterborne routes, as well as through exposure to chicken feces (Butzler, 2004; Kaakoush et al., 2015; Pitkänen, 2013). Exposure to live chickens has been identified as an important transmission route of *Campylobacter* in studies spanning multiple LMICs (Butzler, 2004). In a recent meta-analysis, exposure to domestic poultry was significantly associated with higher odds of campylobacteriosis (Zambrano et al., 2014). In a study in Lima, Peru, exposure to chickens in the household was strongly associated with *Campylobacter jejuni* infection; no specific foods were associated with *C. jejuni* infection in the same study (Grados et al., 1988). Other research in Lima, Peru reported that children often come into contact with chicken feces, that many chickens are infected with *Campylobacter*, and that *Campylobacter* survives well on the patios of households where children may come into contact with animal feces (Marquis et al., 1990). Having chickens infected with *C. jejuni* was identified as a risk factor for *C. jejuni* detection in children with backyard poultry in Egypt (El-Tras et al., 2015). Transmission of *C. jejuni* between animals (chickens, dogs, guinea pigs, and rabbits) and children was demonstrated in a study in Ecuador, though the children were asymptomatic (Vasco et al., 2016). *C. jejuni* can survive in chicken feces for nearly a week (Ahmed et al., 2013; Kaakoush et al., 2015). Outside of a host, *Campylobacter* survives best in cold, moist environments (Wilson et al., 2008).

Proposed control options include appropriate food preparation and handling, water treatment, and keeping chickens out of the household (though chicken corralling may be ineffective in reducing exposure (Oberhelman et al., 2006)). Providing clean water for livestock can reduce infection in cattle (Ellis-Iversen et al., 2009; Kaakoush et al., 2015).

Since the detection of fluoroquinolone-resistant clinical *Campylobacter* isolates in Africa and Asia in the early 1990s, antimicrobial resistance to *Campylobacter* has become increasingly prevalent in both high-income countries and LMICs, with macrolide resistance also of increasing concern (Luangtongkum et al., 2009).

#### 3.1.2 Non-typhoidal *Salmonella*

The GBD estimates that non-typhoidal *Salmonella* (NTS) causes 90,300 annual diarrhea deaths globally, with 38,500 of these deaths occurring in children under five years old (Wang et al., 2016). Of the diarrheal pathogens considered in the GBD, NTS had the third-highest population attributable fraction for all-age diarrhea mortality, surpassed only by rotavirus and *Shigella* (Wang et al., 2016).

The GBD estimate is limited to diarrheal deaths and does not include deaths from invasive NTS (iNTS), a severe form of NTS with a high case fatality rate that is often not associated with diarrhea/typical NTS presentation (Ao et al., 2015). In 2010, there were an estimated 3.4 million cases of iNTS, with iNTS estimated to cause more than 650,000 annual deaths, approximately half of which occur in Africa (Ao et al., 2015).

Common symptoms of NTS include bloody diarrhea, nausea, and vomiting; other more severe complications (such as hepatomegaly and appendicitis) are less frequently observed (Sánchez-Vargas et al., 2011). iNTS is a major cause of bloodstream infection in Africa (Ao et al., 2015; Feasey et al., 2012). Symptoms of iNTS resemble enteric fever and are life-threatening; they include fever and respiratory complications, often without gastrointestinal symptoms (Gal-Mor et al., 2014). There is a positive correlation between iNTS and HIV, malaria, and malnutrition (Ao et al., 2015). Malaria may also be associated with non-invasive NTS in children (Crump et al., 2015).

NTS has a broad range of animal hosts, including poultry, cattle, swine, and domestic animals, as well as wild animals, reptiles, rodents, and insects (Hilbert et al., 2012). Transmission routes have been studied in developed countries; less is known about transmission where NTS is endemic (Crump et al., 2015; Morpeth et al., 2009). In developed countries, consumption of produce contaminated with animal feces and contact with animals have been described as risk factors (Crump et al., 2015; Williams et al., 2016). NTS can multiply in food and can survive for months in soil, insect feces, or rodent feces (Lynch and Tauxe, 2009; Mitscherlich and Marth, 1984).

Much of the transmission of NTS is considered zoonotic, with little sustained human-to-human transmission (Okoro et al., 2012). However, one study of risk factors for NTS infection in Bangladesh did not find an association between NTS infection and presence of animals in the home (Leung et al., 2013). Foodborne transmission likely accounts for a high proportion of global NTS cases (Majowicz et al., 2010).

Human-to-human transmission has been postulated as more important than zoonotic transmission of iNTS, though it has been demonstrated that *Salmonella* Typhimurium ST313, a dominant cause of iNTS in sub-Saharan Africa, is not host-specific and can infect chickens (Kariuki, 2006; Okoro et al., 2012; Parsons et al., 2013). Other zoonotic serovars of NTS have been associated with invasive NTS infection, e.g., *Salmonella enterica* serovar Choleraesuis, which can be difficult to treat because of antimicrobial resistance (Chiu et al., 2005).

There are more than 2,500 serovars of *Salmonella*; however, only a small subset of these serovars (especially serovars Typhimurium and Enteritidis) are of importance to human health (Crump et al., 2015; Hendriksen et al., 2009).

Improvements in safe handling of food and reductions in malaria prevalence are thought to be effective control measures for reducing NTS; however, evidence on control options for LMICs remains limited, as little data exists on risk factors for endemic transmission (Crump et al., 2015). An NTS vaccine has proved efficacious in poultry; however, there is no vaccine for other animal hosts or for humans (Desin et al., 2013; Gal-Mor et al., 2014). While antibiotics are recommended for invasive disease and for non-invasive disease in elderly and immunocompromised individuals, antibiotics can be harmful and prolong NTS symptoms in previously healthy individuals (Gal-Mor et al., 2014). The prevalence of antibiotic resistance in NTS has been increasing in the past decades and is of public health concern; this topic has been reviewed elsewhere (Crump et al., 2015).

#### 3.1.3 Lassa virus

The United States Centers for Disease Control and Prevention (CDC) reports that surveillance for Lassa fever is not well established. The best current estimates suggest there are 100,000–300,000 cases of Lassa fever annually, resulting in approximately 5,000 deaths, almost all in West Africa (CDC Viral Special Pathogens Branch, 2015). Earlier reports estimated 2–3 million annual Lassa virus cases, resulting in 5,000–10,000 annual deaths (Fichet-Calvet and Rogers, 2009; Saluzzo and Dodet, 1999). Many people infected with Lassa virus are asymptomatic, but symptoms can be severe, resulting in high mortality rates for children and pregnant women. Lassa virus may present similarly to Ebola virus, with common symptoms including fever, headache, sore throat, vomiting, and bleeding (Richmond and Baglole, 2003).

The reservoir for Lassa virus is multimammate rats (*Mastomys natalensis*), which are found in sub-Saharan Africa (Bonwitt et al., 2017). Lassa fever is predominantly identified in West Africa, where rodents may enter the household or food storage area, and where rodents are hunted and consumed (CDC Viral Special Pathogens Branch, 2015; Richmond and Baglole, 2003; Yun and Walker, 2012). People may become infected upon ingestion or inhalation of rodent feces, or by ingesting rodent meat. Person-to-person transmission is also possible, including through sexual transmission, though the contribution of this latter transmission route is unknown (Richmond and Baglole, 2003).

Food hygiene measures, such as keeping rodents from entering food storage areas, have been recommended for Lassa virus prevention (World Health Organization, 2017a). Isolation of patients may reduce spread in health care facilities; coupled with surveillance and contact tracing, this may help curb epidemics (World Health Organization, 2017a). Vaccine research has been proposed, though problems may arise with delivery, as vaccine uptake is already low in the regions most affected (Richmond and Baglole, 2003). In a 2017 meeting, the World Health Organization (WHO) identified Lassa fever as a priority disease for increased focus, based on high epidemic potential with few existing medical countermeasures (World Health Organization, 2017b).

#### 3.1.4 *Cryptosporidium* spp.

*Cryptosporidium* causes an estimated 64,800 diarrhea deaths annually, almost all in children under five years old (Wang et al., 2016). The GBD ranks *Cryptosporidium* second in diarrheal pathogens to which the most deaths in children under five are attributed (Wang et al., 2016). In another burden analysis based on GEMS data, *Cryptosporidium* was estimated to contribute to approximately 200,000 annual deaths in children under two years old in sub-Saharan Africa, India, Pakistan, Bangladesh, Nepal, and Afghanistan (Sow et al., 2016).

*Cryptosporidium* causes acute watery diarrhea, and can cause vomiting and fever (Heymann and American Public Health Association, 2015). *Cryptosporidium* is also significantly associated with prolonged and persistent diarrhea (Baqui et al., 1992; Cruz et al., 1988; Mølbak et al., 1993; Moore et al., 2010) and with growth faltering, the deficits of which may not be recovered by children infected in infancy (Checkley et al., 1998). GEMS identified *Cryptosporidium* as a major cause of moderate-to-severe diarrhea (MSD) in young children, with an increased risk of death among toddlers aged 1–2 years (Kotloff et al., 2013). MAL-ED research further implicated *Cryptosporidium* as a significant contributor to community diarrhea in infants (Platts-Mills et al., 2015). *Cryptosporidium* infection is particularly harmful for immunocompromised individuals (Hunter and Nichols, 2002).

*Cryptosporidium* infection occurs through waterborne, foodborne, person-to-person, and zoonotic transmission routes. *Cryptosporidium* has been identified in more than 150 mammalian species (Fayer et al., 2000), as well as in birds, reptiles, fish, and amphibians (Fayer, 2004). *Cryptosporidium hominis* is transmitted from person-to-person (with the only non-human reservoir being primates), whereas *Cryptosporidium parvum* has a number of animal hosts, predominantly ruminant animals (Cacciò et al., 2005). The two most common species of *Cryptosporidium* found in humans are *C. parvum* and *C. hominis* (Cacciò et al., 2005; Huang et al., 2004) and other important species identified in children in developing countries include *Cryptosporidium canis, Cryptosporidium meleagridis, Cryptosporidium felis*, and *Cryptosporidium muris* (Cama et al., 2008; Xiao and Feng, 2008; Xiao and Ryan, 2004), which are generally considered dog, avian, cat, and rodent species, respectively. While it is thought that anthropogenic strains of *Cryptosporidium* predominate in LMICs, zoonotic strains are still commonly isolated in both symptomatic and asymptomatic individuals in LMICs (Gatei et al., 2006; Xiao et al., 2001; Xiao and Feng, 2008).

One study in Cambodia found an association between having birds in the household and *Cryptosporidium* infection in children (Moore et al., 2016). A study among HIV/AIDS patients in Kenya found a significant positive association between contact with farm animals and *Cryptosporidium* infection (Wanyiri et al., 2014). A study in Ghana did not, however, find an association between presence of animals and *Cryptosporidium* infection among children (Adjei et al., 2004). Research in rural India suggests that animals may play a role in contaminating water sources with *Cryptosporidium* oocysts (Daniels et al., 2016, Daniels et al., 2015).

*Cryptosporidium* has a low infectious dose: even a small number of oocysts can cause symptomatic infection in humans (Xiao and Ryan, 2004). *Cryptosporidium* oocysts are shed in large quantities by infected humans and animals and are immediately infectious upon shedding, contributing to ease of transmission (Dillingham et al., 2002). *Cryptosporidium* oocysts can persist for months in the environment (Fayer, 2004; Xiao and Ryan, 2004).

Control options include boiling or filtering water. If filtering, the pore size must be adequately small to capture the relatively small oocysts (Fayer et al., 2000). Chlorine water treatment alone is not effective for elimination of *Cryptosporidium* because the oocysts are highly chlorine tolerant (Dillingham et al., 2002). Access to sanitation and handwashing, especially for infected individuals and those in contact with livestock, are thought to be meaningful prevention/control measures (Heymann and American Public Health Association, 2015).

#### 3.1.5 *Toxoplasma gondii*

As of 2013, there was very little information on the overall burden of disease of toxoplasmosis (Hotez et al., 2014); however, the burden of congenital toxoplasmosis (CT) alone is estimated to be 1.2 million DALYs annually, resulting from approximately 200,000 cases (Torgerson and Mastroiacovo, 2013). CT occurs in infants after their mothers become infected during pregnancy (Robert-Gangneux and Darde, 2012).

The majority of cases of toxoplasmosis are likely asymptomatic (Robert-Gangneux and Darde, 2012), though case presentation can include a wide range of symptoms including fever, lymphadenopathy, headaches, and visual impairment (Hill and Dubey, 2002). Symptoms are often more severe and life-threatening in the immunocompromised (Robert-Gangneux and Darde, 2012). CT can result in miscarriage, or in visual impairment or mental retardation in children whose mothers were infected during pregnancy (Batz et al., 2013). *T. gondii* infections have been linked with schizophrenia and other psychiatric conditions (Batz et al., 2013).

Cats are the definitive hosts of *T. gondii*. A range of other animals (hundreds of species, including cattle, pigs, sheep, chickens, and other mammals and birds) can serve as intermediate hosts (Robert-Gangneux and Darde, 2012). Transmission can occur through contaminated food, water, or soil (Heymann and American Public Health Association, 2015). Drinking unfiltered water has been associated with toxoplasmosis infection (Bahia-Oliveira et al., 2003). Direct contact with cat feces is also a route of exposure. Most non-congenital cases likely occur through ingestion of cat feces (e.g., on contaminated food) or from ingesting tissue cysts in undercooked meat (Dubey, 1996). Cats can shed large quantities of oocysts (Pena et al., 2006). Oocysts found in moist soil or water can be infectious for up to a year after they are shed (Heymann and American Public Health Association, 2015). Oocysts thrive in moist environments, which may explain high prevalence in the tropics. At least one epidemiologic study found a correlation between rainfall and toxoplasmosis infections (Robert-Gangneux and Darde, 2012).

Control options include filtering water, safe processing and handling of meat, washing produce, washing hands after exposure to cat feces or after handling soil, and reducing pregnant women's contact with cat feces (Heymann and American Public Health Association, 2015; Robert-Gangneux and Darde, 2012). Oocysts are resistant to chlorination (Robert-Gangneux and Darde, 2012).

### 3.2 Category II: pathogens with an unquantified burden of disease with potentially important transmission in animal feces

#### 3.2.1 Shiga toxin-producing *E. coli*

The 2015 GBD does not estimate a burden of disease for shiga toxin-producing *E. coli* (STEC). A recent study estimates that STEC is the cause of 2.8 million acute illnesses and 230 deaths annually, which are likely conservative approximations (Majowicz et al., 2014). Despite the available estimate of STEC mortality and prevalence, this pathogen was classified as having an unquantified burden of disease, as a DALY estimate was not available. Surveillance and availability of diagnostics for STEC are lacking in LMICs (Croxen et al., 2013). STEC diagnostics were available for GEMS and MAL-ED; STEC was not detected at any GEMS site (Kotloff, 2017).

STEC can cause mild or bloody diarrhea that is often accompanied by fever and vomiting. Severe complications can include hemolytic uremic syndrome (HUS), end-stage renal disease, and death (Croxen et al., 2013; Majowicz et al., 2014). STEC infections usually resolve within a week and are self-limiting, but there is currently no protocol to prevent development of HUS after an infection (Croxen et al., 2013). General case management techniques for STEC have been proposed and discourage the use of antibiotics (Croxen et al., 2013; Holtz et al., 2009).

Most descriptions of the routes of transmission of STEC come from developed countries. Transmission via contaminated food (especially beef) and water, contact with animals and their feces, and environmental transmission via soil have been recorded (Croxen et al., 2013). Cattle are considered a primary reservoir for STEC (especially O157 strains), and they usually display asymptomatic carriage (Persad and LeJeune, 2014). STEC has also been isolated from a variety of other animals and insects (Croxen et al., 2013). Exposure to ruminant feces is thought to be important to the burden of disease in humans (Croxen et al., 2013; Gyles, 2007). Many STEC isolates carry antibiotic resistance genes (Croxen et al., 2013). Vaccine development is underway, focused both on vaccines for humans and animals (Croxen et al., 2013).

#### 3.2.2 *Toxocara canis* and *Toxocara cati*

*Toxocara canis* and *Toxocara cati* are nematodes carried by dogs and cats, respectively, and are the causative agents of toxocariasis in humans. A 2014 review of the burden of disease from neglected tropical diseases lists toxocariasis as a condition for which limited or no burden information is available (Hotez et al., 2014), despite evidence that seroprevalence is quite high in a number of developing countries (Macpherson, 2013).

Toxocariasis is asymptomatic in many, but can manifest in a number of different symptoms, dependent on which organ the parasite migrates to (Fan et al., 2013). Visceral larva migrans is one of the more common outcomes of repeat *T. canis* infections and can result in a number of symptoms including headache, fever, abdominal pain, vomiting, diarrhea, fatigue, and weight loss (Fan et al., 2013; Macpherson, 2013). Ocular larva migrans is less common and can result in vision impairment (Fan et al., 2013). Exposure to dogs was significantly associated with *Toxocara* seropositivity in children in a study in Sri Lanka (Fernando et al., 2007).

### 3.3 Category III: pathogens with a substantial burden of disease with transmission in animal feces of limited importance, or with insufficient evidence of importance

#### 3.3.1 Enteropathogenic *E. coli*

Enteropathogenic *E. coli* (EPEC) causes an estimated 12,000 global diarrhea deaths annually, almost all in children under five years old (Wang et al., 2016). Symptoms may include diarrhea (which can be watery and/or contain mucus), vomiting, dehydration, and fever (Croxen et al., 2013; Heymann and American Public Health Association, 2015). EPEC can result in persistent diarrhea and a failure to respond to rehydration therapy (Croxen et al., 2013).

There are two pathotypes of enteropathogenic *E. coli*: typical EPEC (tEPEC) and atypical EPEC (aEPEC), with important differences between transmission routes and illness severity; however, these two types are grouped together in estimations of disease burden. While tEPEC is transmitted only by humans, there are animal reservoirs of aEPEC, including dogs, sheep, rabbits, pigs, cattle, and non-human primates (Croxen et al., 2013; Vasco et al., 2016). Transmission of aEPEC between animals (pigs, dogs, and chickens) and children was demonstrated in a study in Ecuador, though the children were asymptomatic (Vasco et al., 2016). The pathogenicity of aEPEC may be low: in some studies aEPEC has been isolated as often in asymptomatic controls as in diarrhea cases (Ochoa and Contreras, 2011). GEMS did not find a significant association between aEPEC and MSD (Kotloff et al., 2013). Some evidence suggests that aEPEC might be more common than tEPEC in developing countries (Croxen et al., 2013; Ochoa and Contreras, 2011). Because the pathogenicity of the strain carried by animals (aEPEC) remains unclear, animal feces may have a limited role (or insufficient evidence of a potentially important role) in contributing to the overall burden of disease from EPEC.

#### 3.3.2 Hepatitis E virus

Hepatitis E virus (HEV) is estimated to cause 26,700 deaths per year (Wang et al., 2016). Generally, the case fatality rate of HEV is low (∼1%); however, case fatality rates in pregnant women are much higher, at around 20% (Dalton et al., 2008). Increased fatality may also occur among those with underlying liver disease (Kamar et al., 2014). Normally, HEV infections are self-limiting, with symptoms including fever, abdominal pain, and vomiting (Kamar et al., 2014).

There are four main genotypes of HEV found in mammals (genotypes 1–4); there is also a fifth genotype found in birds that is not thought to infect humans (Purcell and Emerson, 2008). Genotypes 1 and 2 are transmitted between humans without zoonotic reservoirs, whereas genotypes 3–4 can have animal hosts. Genotype 3 is capable of infecting humans, but is less virulent to humans than genotypes 1 and 2 (Purcell and Emerson, 2008). There is limited evidence of whether genotype 4 infects humans (Dalton et al., 2008). Genotypes 1 and 2 predominantly circulate in developing countries with poor sanitation (Kamar et al., 2014). Genotypes 3 and 4 are mostly thought to infect humans in developed countries, although the distribution of genotype 3 in swine is widespread globally (Dalton et al., 2008). Despite genotype 3′s widespread global prevalence in swine, most reported human infections have occurred in the United States, Europe, and Japan (Purcell and Emerson, 2008). The importance of HEV transmission from swine to humans is thought to differ geographically, being important in eastern and western China, though of lesser importance in central China and India (Kamar et al., 2014).

Pigs appear to be the main animals responsible for zoonotic transmission of HEV to humans (Kamar et al., 2014). Other animals that may have caused human infections include deer, rabbits, and wild boar (Kamar et al., 2014). The proportion of HEV transmission that is zoonotic is unknown; however, consumption of undercooked meat may contribute most to zoonotic transmission (Kamar et al., 2014). A study in rural Bangladesh did not find an association between animal exposure and HEV (Labrique et al., 2013).

The role of animal feces in the transmission of HEV in LMICs was classified as “of limited importance, or insufficient evidence of importance” as anthropogenic genotypes appear to be more common in LMICs and non-human hosts of HEV are limited. However, given the potential for zoonotic transmission and the high prevalence of HEV genotype 3 in swine worldwide, this pathogen is worthy of attention when considering the burden of disease from improperly managed animal feces. Control of swine feces may be an important control measure for vulnerable groups, such as pregnant women, who have a high case fatality rate from HEV (Dalton et al., 2008).

#### 3.3.3 *Ascaris* spp.

Ascariasis is a helminth infection that can contribute to poor nutritional status in humans. While there are few overt clinical symptoms until the passing of worms in feces, there can be serious complications of infection, such as bowel obstruction (Heymann and American Public Health Association, 2015). The GBD estimates that ascariasis is responsible for 2,700 annual deaths and approximately 1 million DALYs (Kassebaum et al., 2016; Wang et al., 2016).

*Ascaris* is transmitted through ova-contaminated soil—either through direct ingestion or through ingestion on produce (Heymann and American Public Health Association, 2015). Humans and pigs are the main reservoirs of *Ascaris*; there is also evidence that dogs may act as reservoirs for human infection (Shalaby et al., 2010). While previously *Ascaris lumbricoides* was thought to be the human form of *Ascaris,* and *Ascaris suum* was thought to be specific to pigs, there is current controversy as to whether these two species should be considered distinct (Alves et al., 2016; Betson et al., 2014). Zoonotic transmission of *Ascaris* has been given much attention recently. There is mounting evidence that *Ascaris* from pigs (usually *A. suum*) can infect humans, although infection via this route may be less probable than infection from humans (Betson et al., 2014). It is thought that human-to-human transmission of *Ascaris* is the main transmission route in developing countries, whereas zoonotic transmission may be more common in developed countries (Betson et al., 2014). A recent large-scale molecular analysis of *Ascaris* from both human and pigs in Europe, Asia, Latin America, and Africa suggested that the isolates from humans and pigs were indeed distinguishable; however, there was evidence of transmission between humans and pigs. While this route of transmission was very common in Europe, it was uncommon (only “sporadic”) in areas where *Ascaris* infections are endemic (Betson et al., 2014). Thus, the role of animal feces in the transmission of *Ascaris* in LMICs was classified as “of limited importance, or insufficient evidence of importance”.

#### 3.3.4 Hookworms *(Ancylostoma ceylanicum)*

Hookworms (*Ancylostoma* spp. and *Necator americanus*) cause anemia and growth shortfalls, though may be asymptomatic in those with light infections. The GBD estimates hookworm infections to be responsible for approximately 1.8 million DALYs, but does not attribute any deaths to hookworm disease (Kassebaum et al., 2016; Wang et al., 2016).

The predominant species of hookworm in humans is *Necator americanus*, which is exclusively transmitted between humans (Tang et al., 2014), and another important species to public health is *Ancylostoma duodenale*, which is also not zoonotic. *Ancylostoma ceylanicum*, however, is zoonotically-transmitted by cats and dogs and may be of public health importance. This species predominates in humans in southeast Asia (Heymann and American Public Health Association, 2015), and in certain settings in this region its prevalence may rival that of *N. americanus* (Inpankaew et al., 2014). *A. ceylanicum* and other zoonotic hookworms may also cause cutaneous larva migrans upon dermal penetration with their larvae (Reichert et al., 2016). Presence of animal feces in the compound was associated with hookworm-related cutaneous larva migrans in Brazilian children (Reichert et al., 2016). Because of the unknown burden of disease specifically from *A. ceylanicum*, and the limited geographic distribution of this species of hookworm, a limited proportion of the global burden of hookworm infection may arise from zoonotic infections, though transmission in animal feces may be of higher importance in specific regions.

### 3.4 Category IV: pathogens with an unquantified burden of disease with transmission in animal feces of limited importance, or with insufficient evidence of importance

#### 3.4.1 *Arcobacter* spp.

*Arcobacter* is a genus of bacteria closely related to *Campylobacter*, but distinguished as its own genus in the 1990s (Collado and Figueras, 2011). There is limited knowledge on the impact of *Arcobacter* on human health, though *Arcobacter butzleri* has been associated with persistent watery diarrhea and has been isolated in diarrheagenic stools in a small number of studies globally, with prevalence as high as 13% in diarrheagenic stools, from a single study in South Africa (Collado and Figueras, 2011; Lehner et al., 2005). Transmission routes of *Arcobacter* are not well understood, though it is thought that humans are infected via contaminated food or water (Collado and Figueras, 2011). Those in the food safety community have identified *Arcobacter* as a potentially serious foodborne hazard (Collado and Figueras, 2011). *A. butzleri* can be found in the stools of animals such as pigs, cattle, and horses (Collado and Figueras, 2011; Lehner et al., 2005).

#### 3.4.2 *Yersinia enterocolitica*

*Yersinia enterocolitica* can cause diarrhea and abdominal pain, often in young children (Heymann and American Public Health Association, 2015). Symptoms can also be more severe and mimic appendicitis (Gupta et al., 2015). *Y. enterocolitica* has been isolated from the feces of a number of animals such as cows, sheep, goats, dogs, monkeys, deer, and rabbits; however, swine are the major reservoir of importance to human health (Rahman et al., 2011; Wang et al., 2009). Foodborne transmission, often through consumption of pork, is thought to be the main source of yersiniosis cases (Bancerz-Kisiel and Szweda, 2015; Bucher et al., 2008). *Yersinia pseudotuberculosis* has been less well described, though may also be acquired through contact with animal feces and may contribute to the burden of yersiniosis (Nuorti et al., 2004). *Yersinia* infections are associated with cooler climates and were not detected in GEMS or MAL-ED (Kotloff, 2017).

#### 3.4.3 *Giardia duodenalis*

The pathogen *Giardia duodenalis,* synonymous with *Giardia lamblia* or *Giardia intestinalis* (Muhsen and Levine, 2012), can give rise to giardiasis in humans, which may manifest in acute diarrhea and can have longer-term effects on malnutrition and malabsorption of nutrients (Anuar et al., 2014; Ortega and Adam, 1997). *Giardia* is often present in asymptomatic carriers; in fact, *Giardia* was significantly negatively associated with MSD in some age groups in GEMS, having been isolated more commonly in asymptomatic controls than children with MSD (Kotloff et al., 2013). In a systematic review and meta-analysis of the association between *G. duodenalis* and diarrhea in children, it was found that *Giardia* was not significantly associated with pediatric acute diarrhea (and in several studies, was negatively associated with diarrhea), though it was associated with persistent diarrhea (Muhsen and Levine, 2012). The association between *Giardia* and markers of environmental enteric dysfunction (EED) was inconsistent in MAL-ED. *Giardia* was positively associated with a measure of increased intestinal permeability, but showed no significant association (and for one marker, a significant negative association) with measures of intestinal inflammation (Rogawski et al., 2017). Higher exposure to *Giardia* was associated with worse physical growth outcomes (length- and weight-for-age) in MAL-ED (Rogawski et al., 2017).

A review of the global burden of disease of *Giardia* is currently underway (Torgerson et al., 2014). Preliminary results of this review suggest that *Giardia* prevalence is high; however, there is limited or no information available on health outcomes that could be used to estimate the DALYs attributable to *Giardia* infections (Torgerson et al., 2014). Estimates of the burden of disease from giardiasis are not given in the 2015 GBD (Wang et al., 2016).

*G. duodenalis* can be found in the feces of humans, livestock, dogs, cats, rodents, non-human primates, and wild animals (Cacciò et al., 2005); however, of the eight known assemblages of *G. duodenalis*, only two (Assemblages A and B) are associated with giardiasis in humans, with Assemblage A more commonly being associated with symptomatic cases (Cacciò, 2015; Feng and Xiao, 2011). Assemblage A has been commonly isolated in animals such as livestock, dogs, and cats, whereas Assemblage B is less commonly found in these mammals (Feng and Xiao, 2011). While Assemblage A infects animals and can give rise to symptomatic giardiasis in humans, animals are more commonly infected with subtype AI whereas humans are mostly infected with subtype AII (Cacciò, 2015). In an analysis of risk factors for *G. duodenalis* infection in Malaysia, close contact with household pets was identified as a significant risk factor for *G. duodenalis* Assemblage A, but not Assemblage B (Anuar et al., 2014). MAL-ED found having a dirt floor and owning chickens to be significantly associated with *Giardia* infection in children under two years old (Rogawski et al., 2017). Being infected with *Giardia* was strongly correlated with being infected with *Campylobacter* in this study, indicating these pathogens may share transmission routes or susceptibility patterns (Rogawski et al., 2017).

While *Giardia* has a wide range of animal hosts, we classified it as a pathogen for which transmission in animal feces is “of limited importance, or insufficient evidence of importance” as evidence is lacking that verifies that zoonotic transmission significantly contributes to the overall burden of disease from *Giardia*. The zoonotic potential of *Giardia* has been described elsewhere in detail and it has generally been concluded that the public health risk posed by zoonotic transmission of *Giardia* may be minimal (Cacciò, 2015; Feng and Xiao, 2011). In their comprehensive review of the zoonotic potential of *Giardia* spp., Feng and Xiao state that “subtyping data accumulated so far do not support a widespread occurrence of zoonotic transmission” (Feng and Xiao, 2011), which is also a conclusion of another review of the zoonotic potential of *Giardia* (Cacciò, 2015). Epidemiologic data also do not support widespread zoonotic transmission, though these data are largely from high-income countries (Xiao and Fayer, 2008).

#### 3.4.4 *Strongyloides* spp.

*Strongyloides stercoralis* is a roundworm that can infect humans when its larvae penetrate the skin. *S. stercoralis* is estimated to infect 30–100 million people, although difficulties in diagnosing infection may lead to underestimates in prevalence and difficulty in assessing the burden of disease (Bethony et al., 2006; Engels and Savioli, 2006; Schär et al., 2013). Possible clinical symptoms include nausea, abdominal pain or discomfort, diarrhea, larva currens, skin eruptions, and weight loss (Olsen et al., 2009; Siddiqui and Berk, 2001; Viney, 2015). *S. stercoralis* can be life-threatening for the immunocompromised (Viney, 2015). *S. stercoralis* can replicate in humans, potentially causing hyperinfection that can last for years (Olsen et al., 2009; Siddiqui and Berk, 2001). Such chronic cases may contribute substantially to morbidity in a way that is yet unmeasured (Engels and Savioli, 2006). The species of *Strongyloides* that infect humans include *S. stercoralis* and *S. fulleborni*. While *S. stercoralis* has a high prevalence in humans and can be transmitted by dogs, *S. fulleborni* is transmitted by primates, has a limited geographic scope, and is a less common human infection (Olsen et al., 2009; Schär et al., 2013; Viney, 2015). It is currently unclear how much of transmission to humans is anthropogenic vs. zoonotic.

## 4 Discussion

**Fig. 2:**
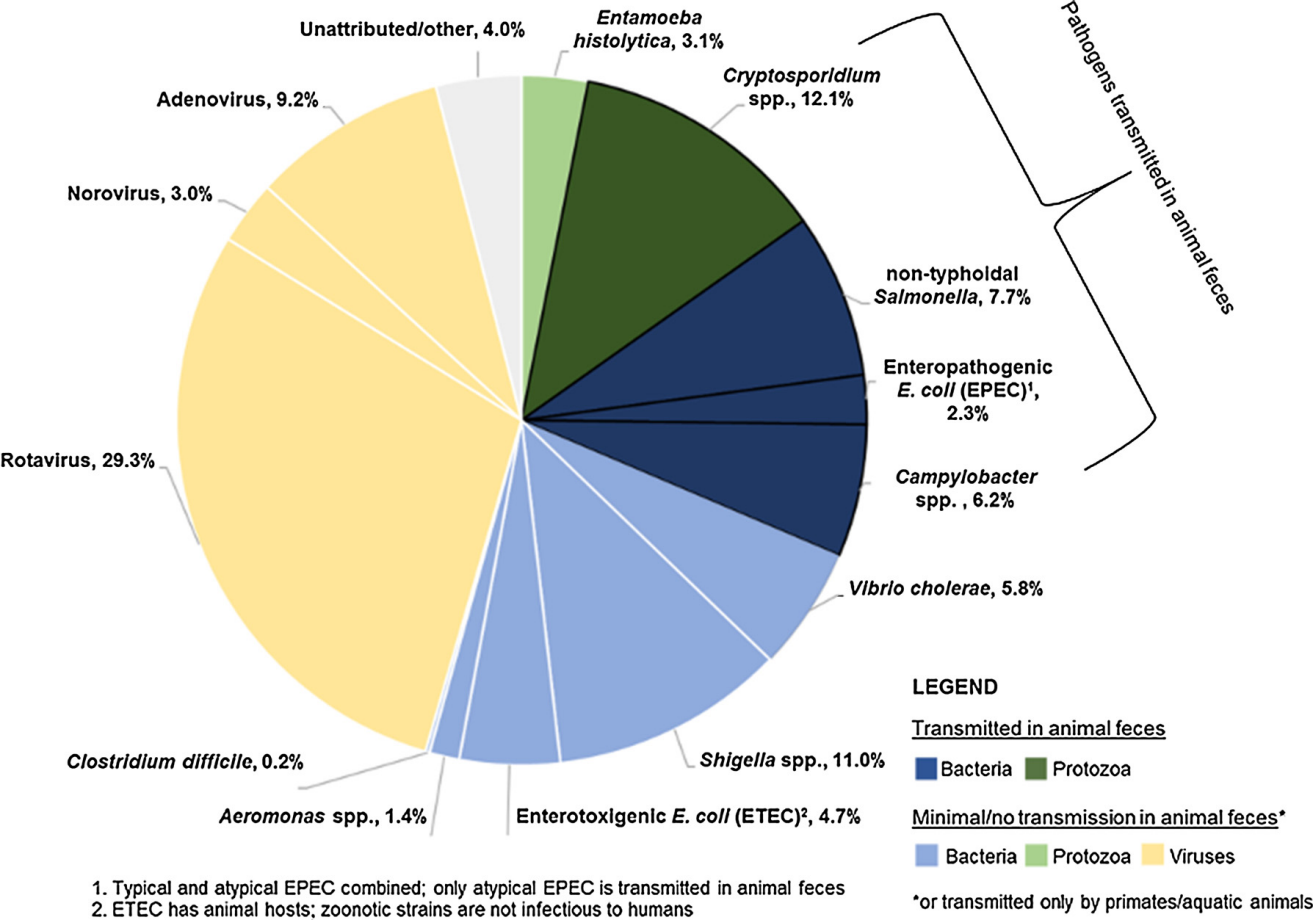
Diarrhea deaths by attributable fraction, children under five years old, Global Burden of Disease Study (2015).

This review compiles evidence on which pathogens may contribute to the burden of disease through transmission in animal feces, which will help prioritize intervention types and regions that could most benefit from interventions aimed at reducing human contact with animal feces. Five pathogens were considered of highest concern based on a substantial burden of disease for which transmission in animal feces is potentially important: *Campylobacter,* non-typhoidal *Salmonella*, Lassa virus, *Cryptosporidium*, and *Toxoplasma gondii*. Combined, these five pathogens cause close to one million deaths annually. The four enteropathogens with GBD diarrhea mortality estimates that have the potential to be transmitted in animal feces (NTS, EPEC, *Campylobacter* spp., and *Cryptosporidium* spp.) are responsible for 28.3% of the ∼500,000 estimated annual global diarrhea deaths in children under five years old (Fig. 2) (Wang et al., 2016). While improvements to water quantity, water quality, and handwashing typically promoted in public health address some secondary transmission routes for both human and animal feces (Penakalapati et al., 2017), a residual burden of disease from these pathogens would be expected even under scenarios of improved sanitation coverage targeted at containing human feces.

While childhood diarrhea, helminth infection, and child growth have been areas of focus in previous reviews of the impact of animal feces on human health (Penakalapati et al., 2017; Zambrano et al., 2014), we found there may be a substantial burden of disease associated with fecal-oral transmission of pathogens found in animal feces but not related specifically to these outcomes (Section 4.2).

### 4.1 Zoonotic origin and intervention approaches

The pathogens that contribute to the burden of disease through their spread in animal feces are found in a broad range of animals (Sections 3.1–3.4). Cattle and other ruminants, poultry and other birds, dogs, cats, and rodents are all hosts of zoonotic pathogens that are carried in feces and capable of infecting humans (Sections 3.1–3.4). Swine also harbor many of the pathogens that contribute to the burden of disease through transmission in animal feces, and are the primary non-human hosts of HEV and *Ascaris suum* (Sections 3.3.2–3.3.3). Many pathogens that can be transmitted in animal feces have also been isolated from horses, amphibians, and insects (Sections 3.1–3.4). Interventions to reduce human exposure to animal feces in LMICs will need to consider this broad range of hosts. Some interventions identified in our review of the impact of animal feces on human health (Penakalapati et al., 2017), such as corralling chickens or providing veterinary care to animals, would not sufficiently cover the range of animal hosts that contribute to the burden of disease by contaminating the environment with their feces.

Relative to other animals, rodents have received little attention in studies addressing exposure to animal feces in LMICs (Penakalapati et al., 2017). Preventing rodents from entering the household and food storage areas could be beneficial in the areas of West Africa where Lassa fever is endemic. Presence of rodent feces in the village compound (though outside of the house) was associated with higher odds of moderate-to-severe diarrhea in a GEMS analysis of animal exposures (Conan et al., 2017).

We found that iNTS contributes significantly to the overall burden of disease represented by the pathogens included in this review, although information is limited on the zoonotic potential of NTS serovars with higher propensity to cause invasive infections. Improved understanding of the transmission routes of serovars of NTS causing invasive disease is needed (Clemens, 2009; Feasey et al., 2012). Additionally, with approximately half of the iNTS deaths and all Lassa fever deaths occurring Africa, this is a continent deserving of attention for interventions aimed at reducing the burden of disease from improperly managed animal feces.

Sanitation programs focused exclusively on containment of human waste may have limited ability to combat illness due to zoonotic transmission of the pathogens identified in this review. Similarly, some water treatment interventions, such as chlorinating water, would do little to reduce the burden from pathogens such as *Cryptosporidium* and *T. gondii* (Section 3.1). The average diameters of *Cryptosporidium* and *T. gondii* ooycsts are approximately 3–5 μm and 10–12 μm, respectively (Isaac-Renton et al., 1998). Though some conventional point-of-use or small-scale water filtration systems may not be adequate for removal of such small oocysts (Jones and Dubey, 2010), the ability of point-of-use water filters to achieve high removal efficacy for *Cryptosporidium* has been demonstrated (Abebe et al., 2015).

A vaccine for Hepatitis E is available in China (Zhang et al., 2015); however, human vaccines are not available for most of the pathogens included in this review. Thus, control efforts will require alternate strategies, including environmental and/or behavioral interventions. Strategies to reduce the burden of disease from pathogens in this review likely need to focus on safe handling/disposal of animal feces; handwashing with soap after contact with animals or animal feces; and safe handling, preparation, and storage of food (Penakalapati et al., 2017). Pathogen infections in infancy and early childhood can have long-term consequences on growth and development (Checkley et al., 1998; The MAL-ED Network Investigators et al., 2014). Exclusive breastfeeding of infants could interrupt foodborne and waterborne transmission of the pathogens of highest concern identified in this review. Interventions should also account for the susceptibility of different individuals to severe sequelae from infections. Many pathogens identified in this review are particularly harmful to pregnant women, the immunocompromised, or those with underlying co-morbidities including malaria, HIV, liver disease, and malnutrition.

### 4.2 Burden of disease associated with pathogens transmitted in animal feces

Improperly managed animal feces contribute to an unknown proportion of the approximately one million annual deaths caused by the four pathogens of highest concern with available death estimates: *Campylobacter*, non-typhoidal *Salmonella*, *Cryptosporidium*, and Lassa virus, with iNTS contributing significantly to this figure. This represents an upper limit of the deaths from these pathogens attributable to improperly managed animal feces globally, as many of these infections may occur through other transmission routes. Contact with animal feces in soil or through other direct routes is important to the transmission of *T. gondii*, which is responsible for 1.2 million annual DALYs from congenital infections as well as an unquantified burden from non-congenital infections (Torgerson and Mastroiacovo, 2013). In addition to these pathogens of highest concern, other pathogens may contribute to the burden of disease from transmission in animal feces in LMICs (Sections 3.2–3.4).

The estimate of one million annual deaths is comprised of ∼200,000 global diarrhea deaths each year from *Campylobacter,* NTS, and *Cryptosporidium* that are captured in the GBD estimates, an additional >100,000 deaths from *Cryptosporidium* above GBD estimates, >650,000 annual iNTS deaths, and 5,000–10,000 annual Lassa virus deaths (Section 3.1). The GBD estimates for diarrheal pathogens capture acute diarrheal deaths, and do not consider deaths that may occur after acute symptoms have subsided, or non-diarrheal deaths caused by the same pathogens. The burden of *Cryptosporidium* in children under two years old only in sub-Saharan Africa and certain regions of Asia exceeds GBD diarrhea estimates by more than 100,000 deaths (Section 3.1.4), when deaths from longer-term follow-up are counted (Sow et al., 2016).

The compiled burden estimates reflect morbidity/mortality from all transmission routes combined. There is limited information on what proportion of the burden of disease caused by these pathogens is attributable to poor management of animal feces in LMICs. The WHO conducted a structured expert elicitation on transmission routes of foodborne hazards (Hald et al., 2016). The study indicated that animal contact is most important to the transmission of *Campylobacter*, NTS, STEC, and *Cryptosporidium* in WHO geographic regions containing LMICs (∼10–20% of transmission), while animal contact was estimated to account for much less of the transmission of pathogens such as *Giardia*, EPEC, and enterotoxigenic *E. coli* (ETEC) (Hald et al., 2016). While this expert elicitation did not specifically identify animal contact as a major transmission route for *T. gondii*, it did find transmission in soil to account for ∼20–40% of *T. gondii* transmission, indicating the importance of animal fecal contamination of soil in the spread of this pathogen (Hald et al., 2016). These findings are consistent with our review, though the WHO focused on direct contact with animals, rather than exposure to animal feces in the domestic environment.

The proportion of pathogen infections that is attributable to contact with animals in the United States has been estimated using outbreak data (Hale et al., 2012), but such estimations are more difficult where pathogens are endemic and surveillance of pathogens found in animal feces is limited. In the absence of an estimate of what proportion of transmission of pathogens in this review was in fact a result of contact with animal feces, we were not able to establish an estimate of the burden of disease resulting from contact with improperly managed animal feces in LMICs.

### 4.3 Human viral enteric pathogens

Viral enteropathogens—which as a group contribute substantially to the burden of childhood diarrhea—are generally host-specific, and we did not find evidence of animal feces contributing to the burden of disease caused by fecal-oral exposure to enteric viruses. One exception is that swine feces can pose a risk for Hepatitis E virus infection, though transmission through this route was considered of limited importance (or insufficient evidence of importance) in LMICs. While animal feces play a minimal or non-existent role in the transmission of some high-burden viral enteropathogens such as rotavirus, there is potential for viral reassortment to occur and potentially change transmission dynamics of such viruses over time (Cook, 2004).

Rotavirus vaccination has been introduced in many countries since 2010, after investigators conducted the diarrhea etiology study that formed the basis of the attribution of diarrheal deaths to various enteropathogens in the GBD (GEMS, conducted 2008–2011). Global rotavirus vaccine coverage is now approximately 25% (PATH, 2016; World Health Organization, 2017c). As rotavirus deaths decline, pathogens such as NTS and *Cryptosporidium* may begin to account for a greater proportion of overall diarrheal deaths.

### 4.4 Limitations

This review considered the role of feces from animals that are common to domestic settings in contributing to the burden of disease; however, uncontained animal excreta can contribute to the burden of disease through other means. Animal feces can contaminate water, which is relevant for some pathogens included in this review (e.g., *Cryptosporidium*), but may also contribute to the transmission of other pathogens such as *Schistosoma* spp., for which animal feces are important in sustaining transmission, even though they do not pose a risk in the household environment (Table 1). Animal feces may also attract flies that can transmit infectious diseases, such as trachoma. Animal urine can transmit pathogens such as *Leptospira* spp., which is responsible for a high number of annual deaths (Costa et al., 2015). Pathogens transmitted only by primates were excluded; however, primates may live in proximity to humans in certain settings (Ghai et al., 2014).

Comparisons of the burden of disease between pathogens were not always possible. Some pathogens (such as the causative agents of echinococcosis and cysticercosis) were excluded based on the comparatively low burden of disease, although the burden from such pathogens could be higher than that of some pathogens that were included in the review with an unquantified burden of disease (e.g., *Arcobacter* spp. or *Yersinia* spp.). Furthermore, estimates of disease burden were based on death or DALY estimates, which may not adequately capture the burden from long-term sequelae such as stunting or EED.

The aggregation of zoonotic and non-zoonotic pathotypes of certain pathogens in the GBD made comparisons difficult between the burden of zoonotic and non-zoonotic species of the same genus. The burden of disease from *Cryptosporidium* spp. is substantial; however, the majority of this burden in LMICs is likely from the transmission of *C. hominis* (for which humans are the primary hosts), which is more prevalent than zoonotic strains of *Cryptosporidium* in many developing countries and may also be responsible for more severe symptoms (Mbae et al., 2015; Xiao and Feng, 2008). Similarly, there is a single estimate for the burden of disease from tEPEC and aEPEC combined, despite only one of these pathotypes being zoonotic.

While the transmission of antimicrobial resistant bacteria and antimicrobial resistant genes from animals to humans is an area deserving of attention when considering the burden of disease resulting from human exposure to animal feces (Dufour et al., 2012), this topic was not specifically considered in this review.

We used a systematic process to identify pathogens for inclusion in this review and to identify research from our team’s prior review of the impact of animal feces on human health. However, when data or information were not identified as part of our systematic search, we conducted a targeted search of the literature to identify missing pieces of information for the review.

### 4.5 Research priorities

While we identify key pathogens that may contribute to the burden of disease through transmission in animal feces in LMICs, in this review we were unable to develop an overall estimate of the burden of poorly managed animal waste on human health. Yet doing so would provide important guidance for policy makers and practitioners for the control of acute and chronic sequelae of enteric infection such as diarrhea, EED, and stunting. Estimates of what proportion of the burden of disease from the pathogens listed in this report is a result of zoonotic transmission would be required to produce such an estimate.

Identifying the proportion of infections with the pathogens considered in this review that are attributable to zoonotic transmission is a key research need. Microbial source tracking methods and genome sequencing of human, animal, or environmental samples present an opportunity to attribute pathogen infections to a human or zoonotic source. Such sequencing could provide new information on pathogens lacking evidence on the attribution of zoonotic transmission to the burden of disease, or provide insights on sources of environmental contamination (Schriewer et al., 2015). For example, our recent systematic review found *Giardia* to be the most commonly-reported pathogen outcome in studies assessing the impact of animal feces on human health in LMICs (Penakalapati et al., 2017); however, information on the zoonotic potential of *Giardia* remains limited for LMICs (Xiao and Fayer, 2008). Though the current state of the literature does not suggest animal feces to be of potentially high importance for *Giardia* transmission, epidemiologic studies and further investigations on host specificity of *Giardia* assemblage subtypes in LMICs could be useful in furthering the evidence on the zoonotic potential of *Giardia* (Feng and Xiao, 2011; Xiao and Fayer, 2008).

Quantifying concentration and shedding rates of pathogens found in animal feces, survival of these zoonotic pathogens in the environment, factors influencing fate and transport of such pathogens, dose responses to these pathogens, and human ingestion/contact patterns with animal feces is important to parameterizing quantitative microbial risk assessment (QMRA) models that could be used to estimate the burden of disease associated with human exposure to pathogens transmitted in animal feces (Dufour et al., 2012). While QMRA methods have been used to assess health impacts from exposure to animal feces in high-income countries (e.g., Soller et al., 2010), we found little evidence of application of this methodology for exposure to animal feces in LMICs; however, identifying such research was not the main focus of the review and it is possible that we have overlooked some work in this area. Metrics such as concentration of common enteric pathogens in the feces of livestock, swine, and poultry (Graham and Nachman, 2010), as well as other domestic and wild animals (Cox et al., 2005), have been estimated in high-income countries and could be used to parameterize QMRA models. Such concentration estimates are available for most of the pathogens of highest concern identified in this review (*Campylobacter, Salmonella*, and *Cryptosporidium*) for high-income settings (Graham and Nachman, 2010). Although utilizing data from high-income countries would provide an opportunity to conduct QMRA modelling in LMICs, estimations of key QMRA parameters specifically for LMICs would be useful given differing conditions in these settings. LMICs have geographically unique and understudied pathogens, as well as vulnerable populations with varying susceptibilities to severe sequelae from pathogen infections (Section 4.1). Estimating these parameters and conducting QMRA analyses for LMICs is a key research priority.

### 4.6 Conclusions

Through this review, we identified key pathogens that may substantially contribute to the global burden of disease in humans through their transmission in animal feces in the domestic environment in LMICs, and several other pathogens that should continue to be monitored for their potential contribution to human illness associated with contact with animal feces. This review fills an important gap in assessing which pathogens may have the potential to substantially contribute to the burden of disease through transmission via animal feces in the household environment in LMICs, and reviews burden estimates for these pathogens. Improved understanding of which pathogens are transmitted in animal feces and the burden from these pathogens provides insight into the residual burden of disease that may be attributable to fecal contamination in the environment under improved human sanitation scenarios in the absence of animal feces control, and can elucidate meaningful ways to reduce the burden of disease via the safe management of animal feces.

## Role of the funding source

This work was supported by the Bill & Melinda Gates Foundation (grant OPP1157522 to Emory University). Karen Levy is supported by the National Institute for Allergy and Infectious Diseases, NIH (grant 1K01AI103544). The content is solely the responsibility of the authors and does not necessarily represent the official views of the funders.

## Conflicts of interest

None.
